# Tobamoviruses have probably co-diverged with their eudicotyledonous hosts for at least 110 million years

**DOI:** 10.1093/ve/vev019

**Published:** 2015-12-16

**Authors:** Adrian J. Gibbs, Jeffrey Wood, Fernando Garcia-Arenal, Kazusato Ohshima, John S. Armstrong

**Affiliations:** ^1^Emeritus Faculty, Australian National University, Canberra, ACT 2601, Australia,; ^2^Statistical Consulting Unit, Australian National University, Canberra, ACT 2601, Australia,; ^3^Centro de Biotecnología y Genómica de Plantas (UPM-INIA) and E.T.S.I. Agrónomos, Campus de Montegancedo, Universidad Politécnica de Madrid, Madrid, Spain and; ^4^Laboratory of Plant Virology, Department of Applied Biological Sciences, Faculty of Agriculture, Saga University, 1-banchi, Honjo-machi, Saga 840-8502, Japan

**Keywords:** tobamovirus evolution, plant evolution, co-divergence, phylogenetic congruence

## Abstract

A phylogeny has been calculated by maximum likelihood comparisons of the concatenated consensus protein sequences of 29 tobamoviruses shown to be non-recombinant. This phylogeny has statistically significant support throughout, including its basal branches. The viruses form eight lineages that are congruent with the taxonomy of the hosts from which each was first isolated and, with the exception of three of the twenty-nine species, all fall into three clusters that have either asterid or rosid or caryophyllid hosts (i.e. the major subdivisions of eudicotyledonous plants). A modified Mantel permutation test showed that the patristic distances of virus and host phylogenies are significantly correlated, especially when the three anomalously placed viruses are removed. When the internal branches of the virus phylogeny were collapsed the congruence decreased. The simplest explanation of this congruence of the virus and host phylogenies is that most tobamovirus lineages have co-diverged with their primary plant hosts for more than 110 million years, and only the brassica-infecting lineage originated from a major host switch from asterids to rosids. Their co-divergence seems to have been ‘fuzzy’ rather than ‘strict’, permitting viruses to switch hosts within major host clades. Our conclusions support those of a coalesence analysis of tobamovirus sequences, that used proxy node dating, but not a similar analysis of nucleotide sequences from dated samples, which concluded that the tobamoviruses originated only 100 thousand years ago.

## 1. Introduction

Knowledge of a pathogen’s origin(s) and its rate of evolution provides a crucial framework for understanding its biology. However, for most viruses, this information can only be inferred indirectly, as viruses leave no fossils and so existing populations provide the only certain information of them. Many recent reports of statistical analyses of the gene sequences of viruses collected on different dates conclude that extant viruses have large mutation rates and must therefore have originated in the past few hundred or thousand years, but doubts about these interpretations (Firth et al. 2010; Sharp and Simmonds 2011; Duchêne, Holmes, and Ho 2014), and clues of much more ancient origins of some viruses, are forcing a re-think. Much of the doubt has arisen from evidence of viruses or virogenes integrated in the genomes of plants (Bejarano et al. 1996; Geering et al. 2005; Chiba et al. 2011; James et al. 2011; Lefeuvre et al. 2011; Lyttle, Orlovich, and Guy 2011; Kondo et al. 2013; Filloux et al. 2015) and animals (Gifford et al. 2008; Katzourakis et al. 2009; Belyi, Levine, and Skalka 2010a, b; Gilbert and Feschotte 2010; Horie et al. 2010; Taylor et al. 2010, 2011; Worobey et al. 2010; Han and Worobey 2012), where the taxonomy of the integrated genes and their ‘hosts’ indicates an ancient origin. Attempts to establish ancient origins by phylogenetic evidence of co-divergence of host and virus (e.g. Wu et al. 2008) have proven to be more controversial (De Vienne et al. 2013). In the tests reported here, we used new methods to re-examine the evidence for and against co-divergence for tobacco mosaic virus (TMV) and its relatives in the genus *Tobamovirus*.

Over the years, several suggestions have been made about when and where tobamoviruses arose, each reflecting the data and methods available at the time. Holmes (1951) was perhaps the first to comment, albeit indirectly. He noted that the *Nicotiana* spp. and other solanaceous plants, that responded to infection by TMV in a hypersensitive manner, are all natives of Central and South America, whereas *Nicotiana* species that are most susceptible to systemic TMV infection are mostly found in North America, southern South America, and Australia. Holmes argued that these differences resulted from the selection of TMV-resistant plant populations in Central and South America, and hence that this region was probably the ‘original habitat of tobacco-mosaic virus in prehistoric times’.

When the idea of a molecular clock was devised (Zuckerkandl and Pauling 1965), it was applied first to the amino acid sequences of the coat proteins (CPs) of the different tobamoviruses known at that time (Gibbs 1980). Their sequence relationships, represented as a Darwinian tree, were found to correlate with their serological relationships. If those viral proteins had evolved at much the same rate as similar proteins of cellular organisms (i.e. cf. 1% per million years), then the differences between them suggested that tobamoviruses had origins as ancient as their plant hosts, which first appeared as fossils more than 100 million years ago. This timing has since been supported by several lines of circumstantial evidence (Gibbs 1999; Gibbs et al. 2008, 2010, 2011), notably the possibility of co-divergence with their long-term eudicotyledonous hosts, because the relationships of the genomes of different tobamovirus species reflects the relationships of their hosts (Lartey, Voss, and Melcher 1996; Gibbs 1999), and as the relevant host clades diverged at least 100 million years ago (Magallón and Castillo 2009; Magallón, Hilu, and Quandt 2013), it suggests that the tobamoviruses did too.

There have however been two recent reports of estimates of the age of the tobamoviruses obtained by Bayesian MCMC coalescence analyses of viral gene nucleotide sequences, and these have produced conclusions about the age of the genus that differ by three orders of magnitude! Pagán et al. (2010) used dated samples to estimate that present-day tobamovirus replicase genes evolve at rates similar to those of most other viruses, and concluded that ‘under these rates, a conservative estimate of the time of origin of the sampled tobamoviruses is within the last 100,000 years, and hence far more recently than proposed assuming co-divergence’. In contrast, Stobbe et al. (2012) inferred the phylogeny of the genus *Tobamovirus* using the same algorithms and found that by ‘calibrating the virus tree with dates of host divergence … resulted in predictions of divergence times of family specific tobamovirus clades that were consistent with the times of divergence of the host plant orders’, which ‘supports the idea that these viruses have co-diverged with their hosts since radiation from a common ancestor’. Stobbe et al. had thus obtained statistical support from, in essence, proxy ‘node dating’ that their phylogeny resulted from an ancient co-divergence, however the internal branches of their phylogeny had poor statistical support.

In this article, we report experiments to resolve the contrasting conclusions obtained by coalescence methods. We used different methods and obtained a tobamovirus phylogeny, that was better supported, especially its internal (basal) branches, and checked its congruence with the hosts’ phylogeny. Our aim was to provide independent support, or otherwise, for co-divergence. We inferred phylogenies by maximum likelihood (ML), to contrast with those obtained by Bayes coalescence and mentioned above, and we used simple procedures to minimize ‘mutational noise’ at the tips of the tree, and thereby increase statistical support for the internal branches of the phylogeny; the concatenated amino acid sequences of all three tobamovirus genes were compared rather than individual gene sequences, and the consensus sequences for tobamovirus species were used rather than sequences from individual isolates.

## 2. Materials and methods

### 2.1 Sequences

Sequences were edited using BioEdit (Hall 1999) and MSWord Gene sequences were sorted, checked, grouped, and duplicates removed using the neighbour-joining (NJ) facility in ClustalX (Jeanmougin et al. 1998), and were then aligned using MAFFT (Katoh and Standley 2013) with its ‘iterative refinement using local pairwise alignment’ option. Wasabi mosaic virus sequences were so close to the youcai mosaic virus population, they were treated as such. The Accession Codes of the 185 sequences in the resulting alignment are given in Supplementary Data Table 1; a few tobamoviruses are represented by single sequences, and the 67 for TMV are the most for a single species. The genomic sequences were separated into their constituent ORFs (i.e. the separate genes for the replicase, movement, and CPs, including any overlapping regions of each gene). They were aligned using the encoded amino acids as guide by the TranslatorX server (Abascal, Zardoya, and Telford 2010; http://translatorx.co.uk) with its MAFFT option. The ORFs were analysed both separately, and in combinations, but mostly as the complete concatenated ORFs (concats) of each genome. The latter were tested for the presence of phylogenetic anomalies using the full suite of options in RDP4 (Martin and Rybicki 2000). Sites in the aligned protein sequences with more than five gaps were removed using POSORT, a DOS command line program available at http://192.55.98.146/_resources/e-texts/README-POSORT.pdf. Consensus protein sequences were derived for each of the species using the ‘Create Consensus Sequence’ function in BioEdit (Hall 1999). This function examines each site in the species alignments and determines whether any amino acid at that site is present at or above the selected frequency and, if one is, records that amino acid in the consensus. When more than one type of amino acid exceeds the selected frequency, then one of those possibilities is chosen at random and recorded in the consensus sequence, and if none exceeds the selected frequency then a gap is recorded.

### 2.2 Phylogenetic analyses

Models for ML analysis were chosen using TOPALi (Milne et al. 2009) and the ProtTest server at http://darwin.uvigo.es (Darriba et al. 2011); the best fit models were found to be GTR+Г_4_ I (Tavaré 1986) for nucleotide sequences and LG+ Г_4_ I (Le and Gascuel 2008) for amino acid sequences. Phylogenetic trees were inferred using PhyML 3.0 (ML) (Guindon and Gascuel 2003), and the support for their topologies assessed using the log-likelihood support for the trees, and the SH-support (Shimodaira and Hasegawa 1999) for their branches.

For comparison the relationships of the 29 tobamoviruses was also calculated from their 100% consensus concatenated amino acid sequences using BEAST v1.8.2 and associated programs (Drummond et al. 2012); the resulting Maximum Clade Credibility (MCC) tree was from a 750,000 cycle analysis using LG + Г_4_ I, a log normal relaxed clock, and constant population size, and the summary tree came from 939 trees obtained from a stable trace after removal of a 250,000 cycle burn-in. The ML and MCC trees were compared by the PATRISTIC method (Fourment and Gibbs 2006).

Trees were drawn using Figtree Version 1.3 (http://tree.bio.ed.ac.uk/software/figtree/), and pairs of trees were compared using PATRISTIC (Fourment and Gibbs 2006).

### 2.3 Comparisons of phylogenies

**Figure 1.**
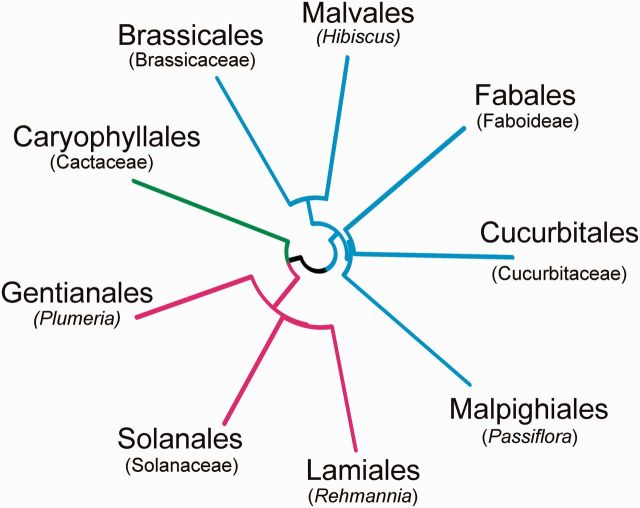
The phylogeny of the primary eudicotyledonous hosts of non-recombinant tobamoviruses. The name of each plant order is given with (in parentheses) the plant taxon which is minimally inclusive of the known primary host species. Asterid orders have red branches, rosid blue, and caryophyllid green. Redrawn from Fig. 1 of Magallón and Castillo (2009).

The congruence of the virus and host phylogenies was assessed using a modified Mantel permutation test (Hommola et al. 2009) to compare their patristic distances. This test ‘explores host–parasite coevolution by testing the null hypothesis that hosts and their associated parasites evolved independently’. Its key feature is the appropriate randomization of the observed data; we used 9,999 permutations. The ‘workspace’ and ‘script’ files for calculating both the correlation coefficient and probability of each comparison using the ‘R environment’ (R Core Team 2013) are available at http://192.55.98.146/_resources/e-texts/blobs/R_files.zip. The plant phylogeny in Fig. 1 was used for the comparisons. It is abstracted from a phylogeny published by Magallón and Castillo (2009) of 265 plant taxa of 52 Angiosperm Orders inferred from the nucleotide sequences of three plastid and two nuclear genes by Bayesian analysis assuming a crown group age of 130 million years. The nine taxa involved in this analysis were measured and converted to Newick format by hand.

A simple pairwise sliding-window method DnDscan (Gibbs et al. 2007), available at http://192.55.98.146/_resources/e-texts/blobs/DnDsCBC.ZIP, was used to identify codons in the alignments that had synonymous, non-synonymous, or no differences, and these codons were separated from the complete sequences using SEQSPLIT v1.0, a Fortran program available at http://192.55.98.146/_resources/e-texts/blobs/SeqSplit.ZIP. NJ trees were calculated from the subsequences using the NJ facility in ClustalX, and were compared by the PATRISTIC method (Fourment and Gibbs 2006).

## 3. Results and discussion

### 3.1 Tobamovirus taxonomy

In January 2014 there were cf. 1,100 tobamovirus gene sequences in the public gene sequence databases, of which 184 were complete tobamovirus genomes that clustered to represent thirty distinct species. The number of sequences representing each virus varied from sixty-seven for TMV to single sequences for twelve of the viruses. The seven genomes of odontoglossum ringspot virus (ORSV) were found to be recombinant when checked with RDP4 (Martin and Rybicki 2000); they had a clear phylogenetic anomaly separating the replicase (RP) from the movement protein (MP) and CP genes, as previously reported by Lartey, Voss, and Melcher (1996) and confirmed by Gibbs (1999), but not by Pagán et al. (2010). The two non-recombinant regions of ORSV sequences were analysed separately with the homologous regions of other tobamovirus genes to reveal their alliances, and were excluded from most of the analyses. There was no other evidence of recombination in the tobamovirus sequences.

Trees of the non-recombinant tobamoviruses were mostly calculated from concatenated genomes (concats) made by reassembling the three aligned gene sequence from each genome. In all the trees the 177 concats of twenty-nine species consistently formed eight lineages with significant statistical support (SH-support mostly >0.9), but the branches linking those lineages had inconsistent support (mean 0.71 SH-support) with no clear consistent pathway of support spanning the basal regions of the trees, which we considered to be the mid 20% of the longest tip-to-tip patristic distances linking the major clusters. The topologies of trees of the individual genes differed from those of the concats in the relative lengths of branches, however the relationships of the genes were mostly similar, and scatter plots of pairwise patristic distances within the trees of the gene trees showed those of the replicase gene and concats were closest (data not shown), which was expected as three-quarters of each concat sequence is that of the replicase.

We therefore used the concat sequences for analysis as they are likely to produce a more representative view of the relationships of the viruses than any of their individual genes, and they separate but conserve both reading frames of the short overlapping regions, with resulting frameshifts, found in some tobamovirus genomes (Goelet et al. 1982; Ryu and Park 1995).

**Figure 2.**
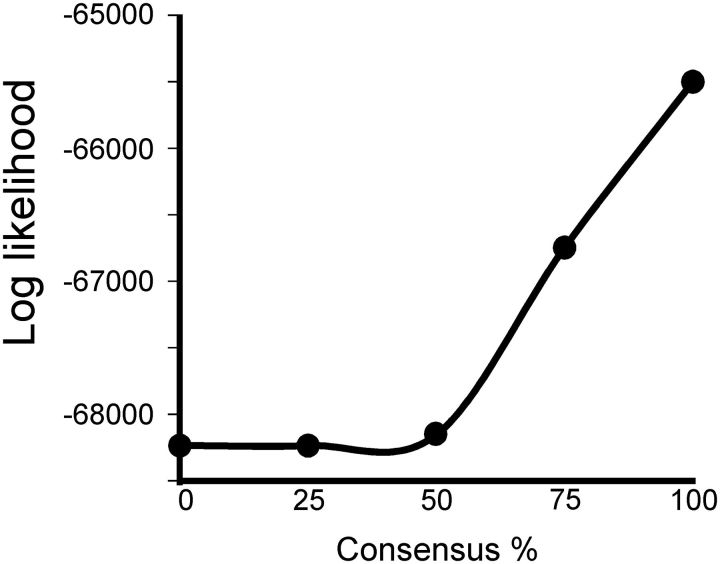
Graph showing the statistical support for ML trees calculated from the aligned twenty-nine concat amino acid sequences obtained using different levels of consensus from 177 tobamovirus genomes.

Three simple strategies were used in an attempt to minimize the ‘mutational noise’ in individual sequences so that the phylogenetic signal from the basal regions of the tobamovirus tree could be detected more easily. First the gene sequences were translated into the amino acid sequences they encoded to remove synonymous codon differences, which are mostly isolate specific. Next, sites in the sequences that included more than five sequences with an alignment gap were removed to minimize the influence of species-specific indels. Finally, consensus amino acid sequences were derived for each of the twenty-nine species using the ‘Create Consensus Sequence’ function in BioEdit (Hall 1999) over the range from 0 to 100 percent consensus. The relationships of the aligned degapped species concats were assessed using PhyML. Trees calculated from the consensus sequences, unlike those calculated from the nucleotide sequences, had a single unbroken statistically significant pathway through their basal regions. Furthermore, the log-likelihood support for individual trees correlated with the threshold level used to obtain the consensus sequences from which each was inferred; the greater the consensus level of the amino acid sequences, the greater the log likelihood support of the tree (Fig. 2).

**Figure 3.**
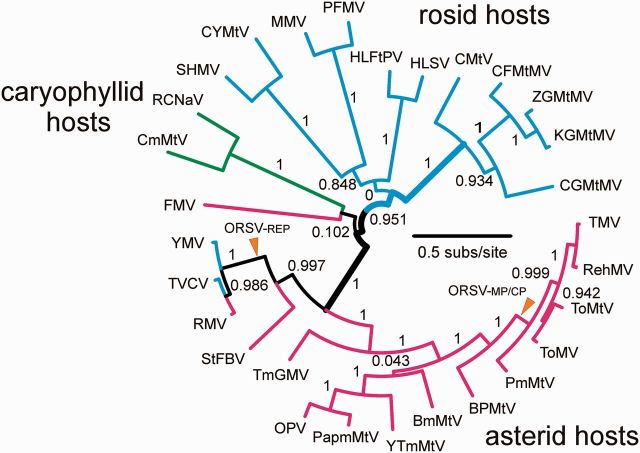
ML phylogeny of twenty-nine tobamoviruses calculated from 100 percent consensus concatenated amino acid sequences of their three main proteins; replicase, MP and CPs. The tree was calculated by PhyML (Guindon and Gascuel 2003) with the LG model (Le and Gascuel 2008). Branches are arranged radially, and are coloured blue, red, or black to show those linked only to rosid, or asterid, or mixed hosts, respectively. The SH-support measure for individual branches is shown, and the branches that have statistically significant support through the base of the tree are bold. The ‘ORSV-REP’ is where the consensus replicase gene of ORSV diverged, and ‘ORSV-MP/CP’ is where its consensus MP/CP concat diverged (see text). Acronyms for the viruses are: BmMtV, *Brugmansia* mild mottle virus; BPMtV, bell pepper mottle virus; CFMtMV, cucumber fruit mottle mosaic virus; CGMtMV, cucumber green mottle mosaic virus; CmMtV, cactus mild mottle virus; CMtV, cucumber mottle virus; CYMtV, *Clitoria* yellow mottle virus; FMV, frangipani mosaic virus; HLFtPV, *Hibiscus* latent Fort Pierce virus; HLSV, *Hibiscus* latent Singapore virus; KGMtMV, kyuri green mottle mosaic virus; MMV, maracuja mosaic virus; OPV, Obuda pepper virus; PapmMtV, paprika mild mottle virus; PFMV, passion fruit mosaic virus; PmMtV, pepper mild mottle virus; RCNaV, rattail cactus necrosis associated virus; RehMV, *Rehmannia* mosaic virus; RMV, ribgrass mosaic virus; SHMV, sunn-hemp mosaic virus, StFBV, *Streptocarpus* flower break virus; TmGMV, tobacco mild green mosaic virus; TMV, tobacco mosaic virus; ToMtV, tomato mottle virus; ToMV, tomato mosaic virus; TVCV, turnip vein-clearing virus; YMV, youcai mosaic virus; YTmMtV, yellow tailflower mild mottle virus; ZGMtMV, zucchini green mottle mosaic virus.

Figure 3 shows the topology and branch support for the 100 percent consensus tobamovirus species tree. The unbroken pathway of statistically significant support spans the basal region of the tree (broader branches: SH-support of 1, 0.951, and 1), and links the top right cluster (CMtV through to CGMtMV) to the bottom twin clusters (YMV to StFBV, and TmGMV through to TMV). The other four smaller clusters (top left) form two well-supported pairs, but their attachment to the statistically supported pathway is undecided (SH-support 0.102 and 0), indicating uncertainty in whether they are linked to it as pairs or separately.

### 3.2 Primary hosts

Tobamoviruses have mostly been named after the host plant from which they were isolated. These are their ‘primary hosts’ (Stobbe et al. 2012), though most tobamoviruses are known to have a wider host range, especially among horticultural plants; TMV, for example, has been isolated from large numbers of dicotyledonous and some monocotyledonous plants (Pfleger and Zeyen 2008), and infects an even larger number of species in experiments. Nonetheless the distribution of primary hosts of the viruses in Fig. 3, as revealed by their names, is strikingly non-random. For instance, all ten of the viruses isolated from species of the Solanaceae fall into the same cluster (bottom right of Fig. 3), and the only non-solanaceous host of that cluster, *Rehmannia*, is from a related family of the Lamiales, as too are *Plantago* and *Streptocarpus*, which are the hosts of two viruses in the sister cluster (bottom left of Fig. 3). Similarly, all the viruses first isolated from cucurbits form a single cluster (top right of Fig. 3), and similarly the viruses isolated from brassicas, cacti, hibiscuses, legumes, and passifloras; each form their own small clusters in the phylogeny, even though the individual members of most pairs were isolated by different virologists from infected plants that were separated from one another and often found on different continents.

The clusters of tobamoviruses defined by virus and primary host taxonomy fall into two mega clusters separated by the basal region of the phylogeny. One includes most of the viruses with rosid primary hosts (blue branches in Fig. 3), and the other, those with asterid or caryophyllid hosts (red and green branches, respectively, in Fig. 3). Notably the caryophyllid branch is long and connected directly to the basal region. The only exceptions are the two brassica-infecting tobamoviruses, which although from rosid hosts form a cluster with those from *Plantago* and *Streptocarpus* (asterids); a clear indication of a likely host switch.

Figure 3 also shows the position (ORSV-REP) where the replicase gene of ORSV joined the tree when its 100 percent consensus sequence is included in an ML analysis of the tobamovirus replicase genes, and likewise the position (ORSV-MP/CP) where the 100 percent consensus MP/CP concat sequences of ORSV joined when they are included in an MP/CP ML analysis.

The congruence of tobamovirus and host taxonomy obtained using the full concat sequences was checked using the much more numerous CP sequences available in Genbank. Seven hundred and sixty-three CP gene sequences were found (January 2015), translated and aligned with the thirty 100 percent consensus CP (CCP) sequences from the concats. In both NJ and ML analyses, 722 (94.6 percent) of the sequences grouped closely with the CCP sequence with the same name, and only thirty-eight (mostly TMVs, ToMVs, or RMVs) grouped closely with CCP sequences with a different name, although all except three of these grouped with known taxa, and seemed merely to be incorrectly named. Three CP genes however appeared to be from phylogenetically distinct tobamovirus taxa; Nigerian tobacco mosaic virus (NTMV; AY137775) was most closely related to, but distinct from TMGMV, tropical soda apple mosaic virus (AY956381) was likewise related to PMMtV, and a distinct CP gene (KF702319) of a CGMMV from luffa was a recombinant of two CGMMV lineages and therefore grouped with neither. Thus, the more numerous CP sequences, which included those of only three additional tobamoviruses, confirm the correlation between primary host taxonomy and tobamovirus taxonomy.

### 3.3 Comparing the topologies of virus and host phylogenies

The topology of the ML phylogeny of twenty-nine tobamovirus species (Fig. 3) was compared with that of their nine primary hosts (Fig. 3) using their pairwise patristic distances in a modified Mantel permutation test (Hommola et al. 2009). The virus and host patristic distances were significantly correlated (coefficient 0.786; *P* = 1e−06). When each of the viruses was removed from the comparisons, one at a time, it was found that removal of data for turnip vein clearing, youcai mosaic, and rehmannia mosaic viruses increased the congruence between host and virus phylogenies most; correlations of 0.828, 0.830, and 0.818, respectively, compared with a mean of 0.781 ± 0.008 for the other twenty-six virus removals), and when the data for all three were removed, the correlation for the twenty-six increased to 0.927 (*P* = 1e−04). Removing the data for more viruses did not significantly increase the correlation. Thus, the topologies of the tobamovirus and host phylogenies are significantly congruent, and this indicates that they have probably co-diverged.

**Figure 4.**
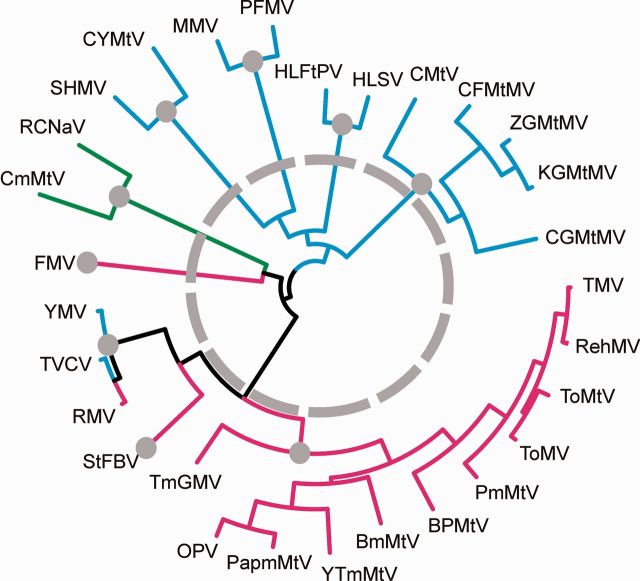
The phylogeny of twenty-nine tobamovirus shown in Fig. 2 with a grey broken circle indicating the basal regions of the tree where part or all of every branch was shortened first to give a branch length corresponding to 0.0001 s/s. The grey disks indicate the positions to which all the basal branches were shortened for a more stringent test of congruence. For an explanation see text.

We also tested whether the existing pattern of tobamovirus and host specificities could have arisen more recently if, for example, an ancestral tobamovirus with diminished host specificity was able, over a short period of time, to infect and adapt to a wide range of plant lineages, but subsequently become adapted and confined to the present primary hosts. To simulate this possibility the tobamovirus phylogeny (Fig. 3) was modified by collapsing (shortening) its internal (basal) branches while maintaining their terminal (distal) regions and branching pattern. Two levels of shortening were tested (Fig. 4). First all basal branches were shortened to the level in the phylogeny where the Solanaceae-infecting and Brassica-infecting tobamoviruses diverged. This removed the differences of branch lengths in the basal half of the tree but, as a result, the original Mantel test correlation of 0.786 (*P* = 1e−06) decreased to 0.705 (*P* = 0.009). In a more stringent test, all lineages were shortened to the basal divergence of each host group (Fig. 4) and, as a result, the correlation decreased to 0.111 (*P* = 0.091). By comparison, when the host:virus linkages were completely randomized, the mean correlation of ten tests was 0.0015 (±0.040) *P* = 0.474 (±0.249). These tests indicate that the congruence between the topologies of host and virus phylogenies involves both the basal and distal regions of the virus tree, and that an ancient host switch is significantly more likely than a recent one.

Finally, we checked whether the algorithm used to infer the virus tree affected the outcome of the comparisons with the host tree. A tree of the twenty-nine tobamoviruses was computed by a Bayesian coalesence method similar to that which Magallón and Castillo (2009) used to obtain the host phylogeny. The Maximum Clade Credibility (MCC) phylogeny we obtained was almost the same as the ML phylogeny; Supplementary Fig. 1 is a graph of the pairwise patristic distances showing how closely similar the two phylogenies are. The modified Mantel permutation test comparisons of the MCC virus and host phylogenies again gave almost the same results; correlation 0.786 for the twenty-nine virus by nine host comparison, increasing to 0.825, 0.825, and 0.822, when the three aberrant viruses were removed one at a time, and 0.921 when all three were removed, but decreasing to 0.755 when the basal branches were shortened, and 0.043 when maximally shortened; all these correlations had *P* ≤ 1e−04 except the final correlation where it was 0.298.

## 4. Conclusions

In this article, we report that phylogenies calculated from consensus amino acid sequences of tobamoviruses show clearer statistical support for the branches of their basal regions than those calculated from the individual nucleotide sequences from which they were translated. These results allow us to discuss with more certainty whether the tobamoviruses have co-diverged with their hosts or have infected and adapted to pre-existing host lineages, because co-diverging viruses and hosts will have trees with similar topology, whereas viruses adapting to pre-existing host lineages will have phylogenies that differ from those of their hosts, as is found with many plant viruses (e.g. potyviruses; Gibbs, Nguyen, and Ohshima 2015). Therefore, the fact that most of the tobamoviruses form three groups of lineages that are congruent with the three groups of eudicotyledonous host plant lineages, asterids, rosids, and caryophyllids, clearly supports the co-divergence conclusion. Furthermore, using a modified Mantel permutation test to compare the phylogenies of nine plant and twenty-nine tobamovirus lineages we obtained strong statistically significant support for the congruence of the host and virus phylogenies, especially when the data for the brassica-infecting tobamoviruses was removed.

It is clear that all the tobamoviruses, except those found in brassicas, have probably co-diverged with their eudicotyledonous hosts, since they formed the asterid, rosid, and caryophyllid lineages around 112.9 million years ago (Magallón and Castillo 2009; Magallón, Hilu, and Quandt 2013). The published conclusion that the tobamoviruses are no more than 100,000 years old (Pagán et al. 2010) is probably incorrect, and is based on the assumption that estimates of evolutionary rates and dates calculated for individual virus species, using isolates collected over a few decades at most, can be extrapolated back into deeper evolutionary time. As virus populations are of finite size, selection or random events will constantly prune the population tree over time, and will ensure that current populations are not a fully representative sample of all the ancient populations. Wertheim and Worobey (2009) stated that coalesence methods give dating estimates that ‘are seriously compromised by unaccounted-for biases’, probably resulting from the combined effects of sequence change saturation and purifying selection (Wertheim and Kosakovsky Pond 2008; Ho et al. 2011; Duchêne, Holmes, and Ho 2014). Furthermore, the evolutionary constraints within stable populations may differ considerably from those that occur during speciation, and/or ‘emergence’ (Gibbs, Nguyen, and Ohshima 2015). Wertheim et al. (2013) suggested that new substitution models must be devised and tested, and we suggest that corroborative studies based on more traditional evolutionary correlations, like those we report in this article, are also required.

**Figure 5.**
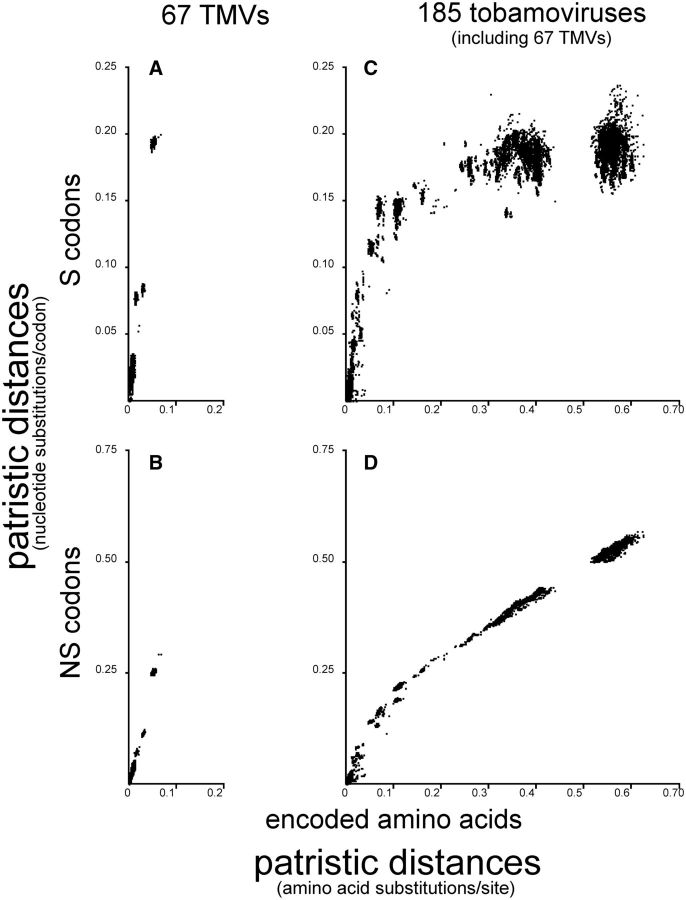
Patristic distance diagrams comparing NJ trees calculated from the S codons and NS codons of sixty-seven TMV concat genomic sequences with NJ trees of the amino acid sequences that the complete sequences encoded.

The influence of mutational saturation is clearly shown when different types of change in the tobamovirus sequences are compared in patristic distance graphs (Fig. 5). One dataset examined was all 185 tobamovirus sequences, and the other was the sixty-seven sequences from the species TMV, that are part of the 185 sequence dataset. For these comparisons, each sequence alignment was separated into two alignments, one consisting of all the codons that only differed synonymously (S changes; Fig. 5A and C), the other the codons that had differed non-synonymously (NS changes; Fig. 5B and D); 51 percent of the codons in the TMV sequences had only S differences and 33 percent were conserved unchanged, whereas in the 185 tobamovirus sequences 92 percent were NS codons and only 7 percent were S. NJ trees calculated from these alignments were then compared by pairwise plots of their patristic distances with NJ trees calculated from the amino acid sequences encoded by the complete sequences. These graphs show the relative rates of accumulation of nucleotide and amino acid changes from the branch tips (bottom left corner) to the base of the tree (top and right sides). Note that if S and NS changes were accumulating at the same rate as the amino acid changes (i.e. the ‘nucleotide clocks’ were synchronized with their ‘amino acid clocks’), then one would expect points in their patristic distance graphs to be clustered along linear diagonals, whereas changes in the relative rates would be indicated by deviation from those diagonals, and a trend asymptotic with the axes would indicate saturation.

Comparisons of the graphs in Fig. 5 show that most initial changes are synonymous changes, but these plateau within individual species in the 185 tobamovirus comparisons at 0.15–0.20 nucleotide substitutions/site (s/s). The differences for all sixty-seven TMV sequences (Fig. 5A and B) are, at most, 0.200 S and 0.293 NS nucleotide s/s and 0.067 amino acid s/s. The clustering of all points >0.25 amino acid s/s in the 185 tobamovirus sequence diagrams (Fig. 5C and D) is broadly parallel to the *x*-axis indicating that S changes mostly saturate by 0.15 nucleotide s/s. In contrast, NS changes (Fig. 5D) show no sign of saturation, and they trend along a diagonal with a separate final cluster comprised of all the distance comparisons linked through the basal branches of the trees. Note that as the S codons mostly vary only in the third nucleotide position, the sites that actually vary in the S codons saturate at around 0.6 nucleotide s/s. In summary then, changes in S codons dominate the nucleotide changes within species but become saturated probably before the amino acid changes exceed 0.3 s/s. As a result, any phylogenetic information they contain is obscured by saturation before they contribute information to the inferred phylogeny of the base of the tree. In contrast, the NS codons, and the changed amino acids they encode, accumulate more slowly than the S changes. They provide phylogenetic information even in the basal branches of the tree of the known tobamoviruses, and show no evidence of saturation.

The tobamoviruses seem to have co-diverged in a ‘fuzzy’ rather than ‘strict’ pattern. This is shown by the tobamoviruses of two plant lineages that are hosts of most (known) tobamovirus species, the Solanaceae and Cucurbitaceae. Over the long term, the tobamoviruses seem to have co-diverged widely within each of these plant taxa rather than tracking the evolution of individual plant species. In the Solanaceae, for example, the genera *Anthocercis* and *Nicotiana* are members of the sub-family Nicotianoideae, whereas *Brugmansia*, *Capsicum*, *Nicotiana*, and *Solanum* (*Lycopersicon*) are from the sister sub-family Solanoideae (Särkinen et al. 2013). These groupings are not congruent with the relationships of the tobamoviruses from these species. Figure 3 shows that those isolated from *Anthocercis* and *Nicotiana* (TmGMV, TMV, and YTmMtV) are scattered among those with primary hosts from the Solanoideae. Similarly, the two most closely related cucurbit-infecting tobamoviruses are nested within a cluster in Fig. 3, but were isolated from species of *Cucumis* (KGMtMV) and *Cucurbita* (ZGMtMV), respectively, whereas the others were all from *Cucumis* species. Fuzziness of co-divergence is also shown by *Rehmannia* mosaic virus, which is clearly a solanaceous tobamovirus, yet was isolated from a species from the Lamiales, which are close relatives of the Solanales. Fuzzy co-evolution could also explain some anomalies in the topology of the base of the tobamovirus tree, as this part of the tree records events when the proto-tobamovirus population was probably infecting a single proto-eudicotyledonous population, for whereas the relationships of the asterid-infecting tobamoviruses and their asterid host families are congruent (*Plumeria* and FMV are sisters to the others), those of the rosid-infecting tobamoviruses and their hosts are not (legumes and cucurbits are the closest related of the rosid hosts). Nonetheless, the two tobamoviruses from cacti (i.e. caryophyllids) are linked in the basal region of the phylogeny to the asterid-infecting frangipani mosaic virus (host *Plumeria*: Gentianales), and this is congruent with recent molecular taxonomies of plants, which place the caryophyllids as a sister group of the asterids rather than the rosids (Magallón and Castillo 2009; Jiao et al. 2012; Zimmer and Wen 2013).

If we accept that the tobamoviruses are at least 110 million years old, then many of the branches in their phylogeny span millions of years, whereas the existing populations of the viruses and their hosts are much more recent. This is clearly shown by the present population of ORSV, a recombinant, which Pagán et al. (2010) showed to have a ‘Time to Most Recent Common Ancestor’ of 43–47 years, yet acquired its genes from two ancestors that diverged from others around 30 million years ago; so there is no evidence from the existing data when, during that long period, the recombination occurred and gave rise to the proto-ORSV. It is also likely that many of the present tobamovirus populations may have spread quite recently from species within their preferred primary host taxon to their current host species, indeed only two of the viruses in this study were isolated from wild plants in their natural environment; passion fruit mosaic virus from *Passiflora incarnata* in the Tallgrass Prairie Preserve of Osage Co., OK, USA (Stobbe et al. 2012), and yellow tailflower mild mottle virus from *Anthocercis littorea* in south west Western Australia (Wylie, Li, and Jones 2014).

Holmes (1951) noted that TMV had clear host links with the Americas, but this feature is not unique to the tobamoviruses that infect Solanaceae. The cacti, from which two tobamoviruses have been isolated, are iconic Central and South American plants, as too is *Plumeria rubra*, the host of FMV, so further sampling for tobamoviruses of these plant groups, and other long-lived endemics of that region, especially ‘core eudicots’, would be of great interest.

## Supplementary data

Supplementary data are available at *Virus Evolution* online.

Supplementary Data Table 1
