# Supplementary material for: Tobamoviruses have probably co-diverged with their eudicotyledonous hosts for at least 110 million years
Source: Virus Evol. 2015 Dec 16;1(1):vev019. doi: 10.1093/ve/vev019 (PMC5014485; doi:10.1093/ve/vev019)
Supplement: Supplementary Data Table 1 [file ve_vev019_index.html]

Supplementary Data | Virus Evolution

## Supplementary Data

files

- Supplementary Data - docx file
