# Supplementary material for: Tobamoviruses have probably co-diverged with their eudicotyledonous hosts for at least 110 million years
Source: Virus Evol. 2015 Dec 16;1(1):vev019. doi: 10.1093/ve/vev019 (PMC5014485; doi:10.1093/ve/vev019)
Supplement: Supplementary Data Table 1 [file Supplementary_Data.docx]

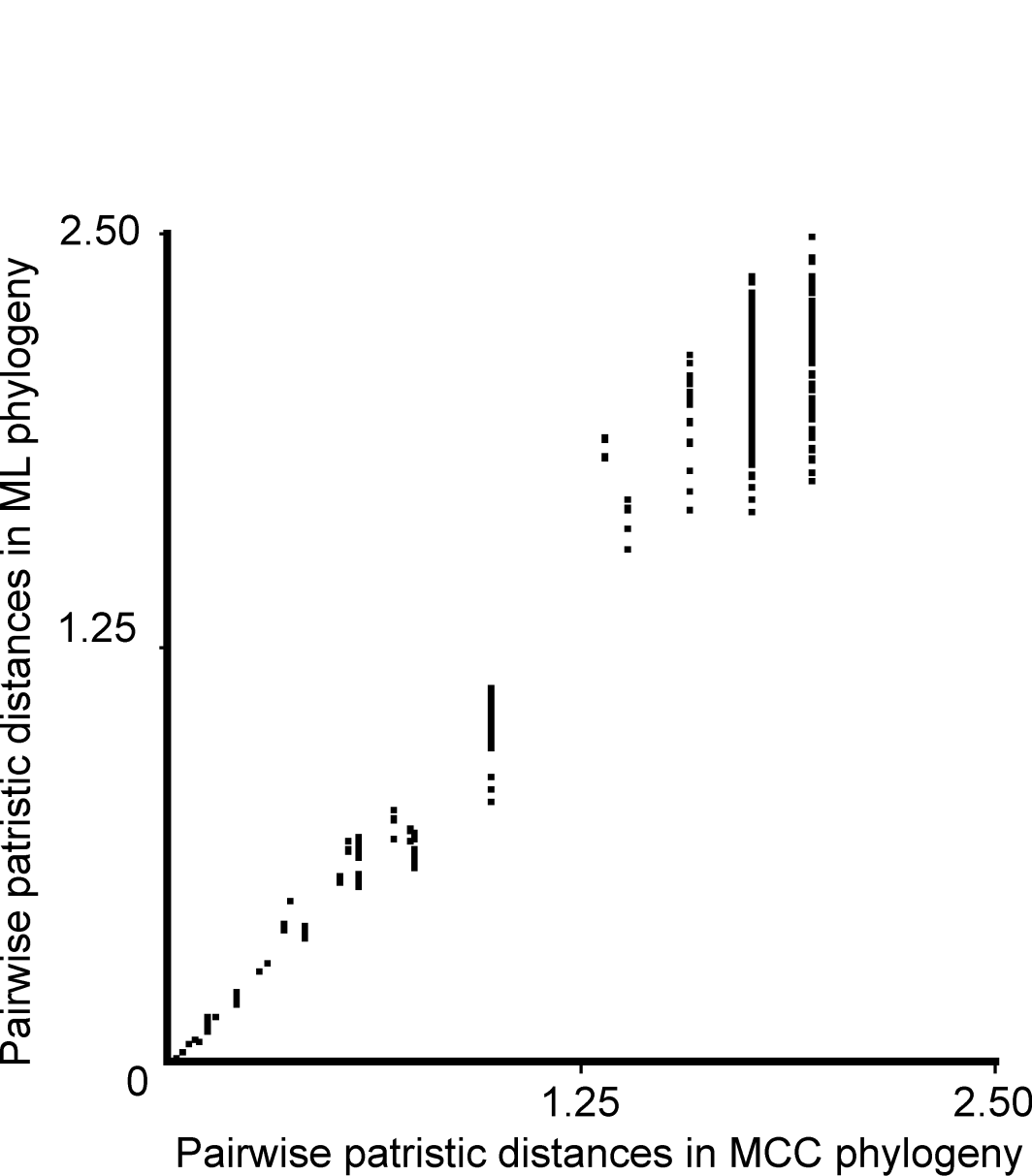


Supplementary Data Figure 1 Graph comparing the phylogenies of the concatenated protein sequences of 29 tobamoviruses calculated by Bayesian coalescence (x-axis) or by maximum likelihood (y-axis).

Supplementary Data Table 1

Viruses, acronyms and the Accession Codes of the sequences analysed:

Bell pepper mottle virus (BPMtV), NC_009642. Brugmansia mild mottle virus (BmMtV), NC_010944. Cactus mild mottle virus (CmMtV), NC_011803. Clitoria yellow mottle virus (CYMV), NC_016519. Cucumber fruit mottle mosaic virus CFMtMV, JN226146, NC_002633. Cucumber green mottle mosaic virus (CGMtMV), AB015146, AB369274, AF417242, AF417243, DQ767631/AY309021/DQ767632/DQ767633/DQ767634DQ767635, EF611826, EU352259, FJ654657, FJ654658, FJ654659, FJ848666, GQ277655, GQ411361, GQ495274, GQ495275, HM008919, HQ692886, KC851866, KC852072, KC852073, KC852074, KF155229, KF155230, KF155231, KF155232, NC_001801. Cucumber mottle virus (CMtV), NC_008614. Frangipani mosaic virus (FMV), HM026454, JN555602, NC_014546. Hibiscus latent Fort Pierce virus (HLFtPV), FJ196834. Hibiscus latent Singapore virus (HLSV), AF395898, NC_008310. Kyuri green mottle mosaic virus (KGMtMV), AB015145, AB162006, NC_003610. Maracuja mosaic virus (MMV), NC_008716. Odontoglossum ringspot virus (ORSV), AY571290, KF855954, NC_001728, S83257, U34586, U89894, X82130. OPV, Obuda pepper virus (OPV), L11665, NC_003852. PapmMtV, paprika mild mottle virus (PapmMtV), NC_004106. Passion fruit mosaic virus (PFMV), JF807914, NC_015552. Pepper mild mottle virus (PmMtV), AB000709, AB069853, AB113116, AB113117, AB126003, AB276030, AB550911, AF254924, AJ308228, AY859497, NC_003630. Rattail cactus necrosis associated virus (RCNaV), NC_016442. Rehmannia mosaic virus (RehMV), AB628188, JX575184, NC_009041. Ribgrass mosaic virus (RMV), GQ401365, HQ667978, HQ667980, JQ319720, NC_002792. Streptocarpus flower break virus (StFBV), NC_008365. Sunn-hemp mosaic virus (SHMV), U47034. Tobacco mild green mosaic virus (TmGMV), AB078435, DQ821941, EF469769, JX534224, NC_001556. Tobacco mosaic virus (TMV), AB369275, AB369276, AF165190, AF273221, AF395127, AF395128, AF395129, AJ011933, D63809, EF392659, FR878069, HE818410, HE818411, HE818412, HE818413, HE818414, HE818415, HE818416, HE818417, HE818418, HE818419, HE818420, HE818421, HE818422, HE818423, HE818424, HE818425, HE818426, HE818427, HE818428, HE818430, HE818431, HE818432, HE818433, HE818434, HE818435, HE818436, HE818437, HE818438, HE818440, HE818441, HE818442, HE818443, HE818444, HE818445, HE818446, HE818447, HE818448, HE818449, HE818450, HE818451, HE818452, HE818453, HE818454, HE818455, HE818456, HE818457, HE818458, HE818459, HE818460, JF920727, JX993906, KF280646, NC_001367, V01408J02415, V01409, X68110. Tomato mosaic virus (ToMV), AB083196, AB355139, AF155507, AJ132845, AJ243571, AJ417701, DQ873692, FN985165, GQ280794, NC_002692, X02144, Z92909. Tomato mottle virus (ToMtV), NC_022230. Turnip vein-clearing virus (TVCV), DQ658743, JN205073, JN205074, NC_001873, U03387/L22518, Z29370. Yellow tailflower mild mottle virus (YTmMtV), KF495565, NC_022801. Youcai mosaic virus (YMV), AB017504, AB254821, AB261175, AY318866, D38444, DQ223770, EU571218, JN634066, JX422022, KF137561, NC_003355, NC_004422, U30944. Zucchini green mottle mosaic virus (ZGMtMV), AJ252189, NC_003878.
